# Adverse Childhood Experiences, Resilience, and Emotional Problems in Young Chinese Children

**DOI:** 10.3390/ijerph20043028

**Published:** 2023-02-09

**Authors:** Yantong Zhu, Gengli Zhang, Tokie Anme

**Affiliations:** 1 Graduate School of Comprehensive Human Sciences, University of Tsukuba, Tsukuba 3058577, Japan; 2 Faculty of Educational Science, Anhui Normal University, Wuhu 241000, China; 3 Faculty of Medicine, University of Tsukuba, Tsukuba 3058577, Japan

**Keywords:** adverse childhood experiences, young Chinese children, resilience

## Abstract

Resilience plays an important role in the relationship between adverse childhood experiences (ACEs) and children’s health. Young children are often neglected in ACEs research and suffer from the negative consequences of ACEs. However, few studies have focused on the relationship between ACEs and emotional problems in young Chinese children and the moderating and mediating effect of resilience on this relationship. This study included young children at the beginning of their kindergarten year (*n* = 874, 42.80 ± 4.09 months) from Wuhu City, China, to examine the mediation and moderation effects of resilience on early-life ACEs and emotional problems. Our results show a positive direct effect of ACEs on emotional problems. Furthermore, a positive indirect effect of ACEs and emotional problems on resilience was found. A moderating effect of resilience was not observed in this study. Our findings (a) highlight the significance of paying more attention to early ACEs and revealing a better understanding of the effect of resilience on ACEs at an early age and (b) indicate that age-specific interventions should be provided to enhance young children’s resilience when exposed to adversity.

## 1. Introduction

Adverse childhood experiences (ACEs) refer to abuse, neglect, and dysfunctional households and can be harmful to an individual’s health and wellbeing [1]. Studies have shown that ACEs have a negative effect on children’s lifelong mental and mental health [2,3]. A new and emerging line of research involves the examination of the effect of early adversities on preschool children’s mental health. Studies in Western countries have emphasized that adverse experiences and trauma occur at particularly high rates in young children [4]. According to Jimenez et al. [2], one fourth to half of preschool-aged children have been exposed a potentially adverse event by the age of four, with exposure rates being greater for children living in poverty [5]. Early adverse experiences raise the risk of young children having poor mental health [6,7], as well as a higher likelihood of continued exposure throughout life [8]. However, few studies have focused on early adverse experiences in Chinese children. Early adverse experiences are underreported among Chinese children, probably because of the lack of a formal child protection system and cultural norms that prevent confession or acknowledgement [9]. Hence, it is critical to examine the negative effects of early adverse experiences on the mental health of young Chinese children.

Although studies have revealed the relationships between early adverse experiences and short-and long-term mental health in children, few studies have explored the underlying mechanisms and protective factors. Resilience refers to the skills which can maintain normal functioning or adapt successfully when facing the adverse experiences or threat [10]. Studies have suggested that resilience may have a mediating or moderating effect on the relationship between ACEs and negative outcomes [11]. Therefore, the purpose of this study is to explore the moderation and mediation effects of resilience on the relationship between ACEs and their outcomes.

### 1.1. ACEs and Emotional Problems among Young Children

Mental health problems in children mainly refer to the most common problems which are anxiety and depression and are frequently comorbid [12]. The appearance of these emotional problems, as well as their frequent comorbidities, has been reported in young children [13]. Studies have suggested that ACEs are an important factor which can cause emotional problems [14]. Previous studies have revealed several potential explanations for the relationship between early ACEs and emotional problems in young children. Children exposed to early adverse experiences were more accurate in identifying threatening stimuli [15], and while this capacity serves an adaptive purpose in hazardous conditions, it could increase the likelihood of mental health disorders over time [14]. The findings also showed that children who have exposure to adverse experiences tend to adopt maladaptive emotional regulation skills such as expressive suppression, disengagement, and rumination and less likely to engage in successful strategies such as cognitive reappraisal [16], which may cause early emotional problems [17]. A recent study revealed that more ACEs were related to more emotional problems among American youths [18]. However, few studies have revealed the relationship between ACEs and emotional problems among eastern children at an early age.

### 1.2. Moderation and Mediation Role of Resilience

Resilience may be crucial in the relationship between ACEs and emotional problems. Research has shown resilience to be a protective mechanism that can propel a person to thrive when they are faced with adverse experiences [19]. Consequently, resilience is assumed to play a protective role in the link between ACEs and emotional problems. Ding et al. [20] found that resilience plays a moderating role in the relationship between childhood adverse experiences and depression among children aged 9–17. Yu et al. [21] revealed that resilience also acts as a moderator between ACEs and mental health symptoms among young adults. However, a non-significant moderating effect of ACEs and emotional problems was found among American youth [18]. The mediating effect of resilience has also been explored in previous studies, which suggest that experiencing adverse experiences may decrease the level of resilience, which in turn may cause more emotional problems [22]. On the contrary, some studies found no mediating effect of resilience factors on adverse experiences and emotional problems [11]. As most studies focus on the effect of resilience in the associations between ACEs and emotional problems from middle childhood to adulthood, few studies have examined whether resilience plays a moderating and mediating role in the relationship between ACEs and emotional problems in young children.

### 1.3. Current Study

Although many studies have explored how ACEs are associated to emotional problems and the role of resilience in these relationships, no studies have revealed the relationship between lifetime ACEs and emotional problems at age three in young Chinese children and the effect of resilience. To expand the current knowledge, this study aims to (1) reveal the relationship between early ACEs and emotional problems at age three, (2) examine whether resilience play a mediating role in the relationship between ACEs and emotional problems, and (3) explore whether resilience moderates the relationship between ACEs and emotional problems.

## 2. Materials and Methods

### 2.1. Participants and Procedures

This study used the baseline year data from the Wuhu Family Study. Wuhu is a prefecture-level city located in central China; the economy status of Wuhu is at a medium national level [23]. From October to December 2022, we randomly invited 11 kindergartens from seven districts in Wuhu City (including rural and urban areas), based on their socioeconomic level and the children population density of the region. Kindergarten principals and teachers were informed of the study objectives. Parents were asked to provide children’s health status, e.g., chronic diseases, allergies, ADHD, prior to the child’s enrollment in kindergartens. After enrollment, the kindergarten organized a physical examination of the child by pediatricians to confirm the child’s physical health. Any children with health problems were excluded from the study. Children whose parents had communication impairments or physical and mental illnesses were also excluded from our study. We invited all eligible newly enrolled children and their parents in the first grade of kindergarten to participate in our cohort study. All parents were made aware of the study’s objectives, methods, and options to withdraw their participation at any time. A total of 950 invitations were sent to the children’s main caregivers, and 874 of them provided consent. After collecting all the consent forms, web-based questionnaires were used though the “WenJuanXing” online survey platform. We then distributed the online questionnaire, which included demographic information and the studied variables. All 874 caregivers provided valid data and that was used for later analysis. This study was approved by the Ethics Committee of Anhui Normal University.

Of the participants, 51.4% (*n* = 449) were male. The majority of reports on the questionnaire were completed by the preschoolers’ mothers (76.5%), while others were reported by fathers (22.8%) and other relatives (0.7%). The preschoolers’ annual family income ranged from below ¥50 k to above ¥300 k, with an average income between ¥100 k and ¥150 k; 95.3% of the families received 50,000 yuan per year as their family income, which showed the economic level of our sample was higher than the Chinese average poor family income level [24]. Table 1 shows the descriptive statistics for all study variables.

### 2.2. Measures

#### 2.2.1. ACEs

The Kaiser-CDC ACE Study questionnaire [1,25] was used to evaluate adverse experiences before the age of three. The main caregivers completed the questionnaire, which contains three categories: abuse, neglect, and household dysfunction. Abuse contained three items (physical, emotional, and sexual abuse). Neglect contained two items (physical and emotional neglect). Household dysfunction contained five items, they were domestic violence, parental mental illness, substance use, parental criminality, and parental separation or divorce. Each item was dichotomized as 1 for “experienced” and 0 for “no experienced”. The total ACEs score ranged from 0 to 10. The ACEs questionnaire has been used for screening early ACEs as reported by parents in both Western countries and China [18,26]. The ACEs questionnaire used in this study show a high internal consistency which was 0.72. 

#### 2.2.2. Resilience

Resilience was evaluated using the Devereux Early Childhood Assessment for Preschoolers, Second Edition (DECA-P2) [27]. Parents (caregivers) or teachers filled out the 38-item DECA-P2 Behavior Rating Scale to assesses internal child protective factors which is essential to social and emotional health and resilience and serves as a screening tool for behavioral issues in children [28]. The DECA-P2 scale contains four subscales: Initiative (nine items, i.e., show confidence in his/her abilities (for instance, say “I can do it!”)), Self-Regulation (nine items, i.e., accept a different option when his/her first choice is unavailable), Attachment/Relationships (nine items, i.e., act in such a way that adults smile or express interest in him/her), and Behavioral concerns (11 items, i.e., touched children or adults in an inappropriate way). A 5-point Likert scale ranging from 0 (never) to 4 (always) was rated for each item. This study used the total score of initiative subscale, self-regulation subscale, and attachment/relationships subscale to measure resilience [29]. Raw scores were calculated into T-scores according to the manual. A higher total protective factor score indicated a higher level of resilience. Parent-reported DECA-P2 has shown good reliability and validity in Chinese children [29]. Cronbach’s α for our sample was 0.95. 

#### 2.2.3. Emotional Problems

We used the Strength and Difficulties Questionnaire (SDQ)-Chinese version in this study [30,31]. Prosocial behavior (five items, i.e., considerate of other people’s feelings), emotional problems (five items, i.e., often complains of headaches), conduct problems (five items, i.e., often complains of headaches), hyperactivity (five items, i.e., easily distracted, concentration wanders), and peer problems (five items, i.e., easily distracted, concentration wanders) were the five subscales of the questionnaire. Each item was rated on a 5-point Likert scale ranging from 0 (not true) to 2 (certainly true). The primary caregivers of the children were given the questionnaire, and they were instructed to answer each question based on their child’s behavior over the previous six months. The emotional problem subscale was used in this study. Totals for the Emotional problems subscale ranged from 0 to 10. The internal reliability of the SDQ in this study was 0.67, and that is consistent with previous studies.

#### 2.2.4. Covariates

Children’s age (months), sex (1 = boys, 2 = girls), and socioeconomic status (SES) were considered as covariates in this study. The SES consisted of five indicators: the father’s occupation, education level, the mother’s occupation, education level, and the family’s annual income. The average standardized score of five indicators was calculated as the family SES in our study [24].

### 2.3. Statistical Analysis

First, we used descriptive and correlation analyses to analysis the studying variable. Second, a mediation model was performed using Hayes PROCESS Macro-Model 4 to explore the mediation effect of resilience on the relationship between ACEs and emotional problems. Third, a moderation model was performed using Hayes PROCESS Macro-Model 1 to explore whether resilience play a moderating role in the relationship between ACEs and emotional problems. A total of 5000 bootstrap samples were used to estimate the 95% confidence intervals for the significance of effects. Age, sex, and family SES were considered as covariates in the data analysis.

## 3. Results

### 3.1. Descriptive Information for Characteristics

In total, 874 children were included in this study. Table 1 shows the descriptive information for the variables. The mean and standard deviation of children’s age was 42.80 ± 4.09 month. Of the total sample, 51.4% (*n* = 449) were boys and 48.6% (*n* = 425) were female. The cumulative ACEs score was 0.42 ± 1.06. Of the children, 22.1% (*n* = 193) experienced at least one type of ACEs. The mean score for resilience was 44.16 ± 10.17. The average emotional problems score was 1.33 ± 1.27. Table 2 shows the bivariate correlations among the main variables.

### 3.2. Mediation Analysis

Table 3 shows the mediating effect of resilience on the relationship between ACEs and emotional problems. The ACEs score (b = 0.12, *p* < 0.01) displayed a significant positive direct influence on emotional problems. The ACEs score was negatively associated with resilience (b = −1.20, *p* < 0.001), which in turn was related to children’s emotional problems (b = 0.03, *p* < 0.001). We found that resilience mediated the associations between ACEs (indirect effect = 0.04, Boot SE = 0.01, 95% CI = [0.02, 0.06]) and children’s emotional problems.

### 3.3. Moderation Analysis

A moderation model was used to examine the relationship between ACEs and emotional problems, with resilience as a moderator. The ACEs were positively associated with emotional problems (b = 0.14, *p* < 0.01). Resilience was negatively related to emotional problems (b = −0.03, *p* < 0.001). However, the interaction effect of ACEs’ resilience was not significant (b = −0.01, *p* = 0.49), implying that resilience did not play a moderating role in the relationship between ACEs and emotional problems.

## 4. Discussion

In line with previous research, current research found at least 22.1% of young children experienced at least one type of ACE, which means children at this age also experience different kinds of adverse experiences and trauma. Resilience significantly mediates the relationship between ACEs and emotional problems. However, a moderating effect of resilience was not observed in this study.

Our findings revealed that early ACEs were related to children’s emotional problems at age three, which was similar to previous studies [18]. Given the significance of toxic stress in changing brain circuitry, it is not surprising that ACEs are associated with poor emotional health [32]. Early exposure to toxic stress can cause amygdala hypertrophy and a hyper-responsive or persistently active physiological stress response, which may later cause emotional problems [33]. Previous studies also suggested children who experienced early adverse experiences were also more likely to recognize threatening stimuli [15], and this attentional bias was related to children’s anxiety symptoms [34]. Furthermore, previous studies also suggested that children exposed to adverse experiences tend to use maladaptive regulation strategies more frequently than children who are not exposed [16]. Therefore, further studies should focus on young children to prevent the detrimental effects of early ACEs.

Our findings add insight into the mediating effect of resilience on the relationship between ACEs and emotional problems. Children exposed to higher levels of ACEs may have lower resilience, which may in turn lead to more emotional problems. Relationships that are stable, safe, and nurturing are thought to be the foundation of children’s resilience [35]. Early adverse experiences can be especially stressful for children because many directly worse the relationship [36]. For instance, children who experienced adverse experiences may cause children to construct unreliable and untrustworthy mental representations of their parents, which can result in the development of insecure and disordered attachments [37]. Moreover, early brain development is especially vulnerable to toxic stress brought on by ACEs, exposure to ACEs in early age may cause can disrupt the developing architecture of the brain and affect an individual’s long-term development of resilience [38]. Studies have suggested that resilience play an important role in development of children emotional regulation and mental health [39]. Lack of resilience have been proved that was associated with more stress, depression, and anxiety [19]. Therefore, presence of ACE, along with a lack of resilience, leads to increased emotional difficulties [17].

Contrary to previous studies, no moderating role of resilience in the relationship between ACEs and emotional problems was found. Maat et al. [40] also did not find a moderating effect of resilience on the relationship between early-life stress and behavioral problems. In the study of resilience, there is growing consensus that protective variables may not be universal [40]. The developmental period, the population under research, and the stress experienced by an individual can all influence whether certain factors are protective [11]. Resilience can be regarded as an individual-, family-, and community-level resilience factor [11]. Individual-level resilience was measured in both the current study and that of de Maat et al. [40]. Because young children are going through a rapid period of emotional and physical development, their individual-level resilience may not be able to cope with difficulties, and they are highly reliant on their primary caregiver to provide them with physical and emotional protection. Therefore, traumatic events can be particularly distressing for them and put them at even greater risk of adverse psychological outcomes [41,42]. Recent studies have found that family functioning and regularity can buffer the effect of cumulative risk on internalizing and externalizing problems in early childhood [43]. A secure attachment to caregivers and positive parenting strategies may help children overcome the negative effects of early ACEs [38]. Quality early care and education programs can also create an environment for protective factors to promote positive development among young children with ACEs [44].

Our findings also found the significant associations between covariates (i.e., gender, SES) and resilience. Similar to previous research, we found that girls tend to show the higher level of resilience compared with boys [45]. When exposure to adverse experiences, girls are more likely than boys to utilize resilience factors such as seeking and receiving help [46]. Girls also show higher levels of self-regulation which consider as an important component of resilience than boys during early childhood [47]. We also found a positive relationship between children’s age and resilience, which is consistent with previous studies [48]. Resilience as a dynamic developmental process increases over time as it interacts with the outside world [48]. Furthermore, our findings revealed that children with high family SES were more likely to have high level of resilience and low level of ACEs. According to Wister’s resilience model [49], children with high family SES have greater social and economic resources available to them. In high SES family, parents were more like to use positive parenting strategies and develop security parent-child attachment, which can improve children’s early resilience [48]. Socioeconomic status is a significant contextual factor that has been associated to ACE exposure [49]. Parents from high SES family tend to provide better home rearing environment and less child maltreatment, which can promote resilience in early childhood [35].

This study extended previous research by examining the mediating and moderating effects of resilience on the relationship between early ACEs and young children’s emotional problems. Our study revealed the positive direct effect of ACEs on emotional problems and their mediation, but not the moderating role of resilience in this relationship. Furthermore, our results highlight the importance of screening early for ACEs for children’s mental health, and more interventions related to the family level and early care and education program level should be used for children who are exposed to ACEs in early childhood.

Nonetheless, this study had some limitations. First, all information, such as the ACEs, was reported by parents. Although the fact that retrospective and prospective ACEs reports have converged [36,50], due to the length of time that has elapsed between the events and remembrance, retrospective parents’ reports of early adversities may still be less reliable and may cause retrospective bias and social desirability biases [51]. Second, although the 10-item ACEs questionnaire has been the most frequently used ACEs questionnaire, it may lack some important categories of ACEs, such as financial problems, community violence, and peer victimization [52]. Therefore, future studies should include these ACEs. Third, despite the fact that early childhood is thought to be an opportune period to investigate resilience and adopt early intervention strategies aimed at altering the trajectory of pathways that contribute to the appearance of mental health disorders [53], and DECA-P2 is a reliable tool for screen young children’s resilience [27,28], resilience is still a complex variable to access (i.e., personal-level resilience, family-level resilience) in early childhood. Therefore, we suggest that future study can examine different aspects of resilience. Finally, this cross-sectional study examined the role of resilience in the relationship between ACEs and emotional problems. As the core characteristic of ACEs is the accumulation of cumulative risk over time, retrospective and cross-sectional studies of ACEs may be unable to account for or adequately express the unremitting nature of an ACE [54]. Resilience may also change over time [55]. A longitudinal study could better illustrate the relationship between ACEs, resilience, and young children’s outcomes.

## 5. Conclusions

Our findings revealed a positive association between ACEs and emotional problems among young children and the mediating, but not moderating, role of resilience in this relationship. The results highlight the need for more attention to be paid to early ACEs and a better understanding of the role of resilience at an early age. Individual, family, and community-level interventions should be used to enhancing children’s resilience to adversities at an early age.

## Figures and Tables

**Table 1:** Descriptive information for all studying variables.

Variables	Category	*n* (%) or Mean ± SD
Child’s age (month)		42.80 ± 4.09
Gender	Boy	449 (51.4)
	Girl	425 (48.6)
Father’s occupation	Farmer, unemployed, and non-technical worker	28 (3.2)
	small business owner and semi-technical employee	176 (20.1)
	Worker in technology and semi-pro	251 (28.7)
	Professional, executive, and mid-sized business owner	249 (28.5)
	High-level professional and administrators	170 (19.5)
Mother’s occupation	Farmer, unemployed, and non-technical worker	180 (20.6)
	small business owner and semi-technical employee	101 (11.6)
	Worker in technology and semi-pro	242 (27.7)
	Professional, executive, and mid-sized business owner	289 (33.1)
	High-level professional and administrators	62 (7.1)
Father’s education level	Elementary or lower	3 (0.3)
	Middle school or lower	85 (9.7)
	High school or vocational secondary school	118 (13.5)
	Vocational college degree	217 (24.8)
	Bachelor’s degree	359 (41.1)
	Master’s degree or above	92 (10.5)
Mother’s education level	Elementary or lower	6 (0.7)
	Middle school or lower	78 (8.9)
	High school or vocational secondary school	123 (14.1)
	Vocational college degree	234 (26.8)
	Bachelor’s degree	357 (40.8)
	Master’s degree or above	76 (8.7)
Family’s annual income	<50,000 RMB	41 (4.7)
	50,001–100,000 RMB	148 (16.9)
	100,001–150,000 RMB	207 (23.7)
	150,001–300,000 RMB	325 (37.2)
	>300,000 RMB	153 (17.5)
ACEs		0.42 ± 1.06
0		681 (77.9)
1		115 (13.2)
2–3		46 (5.3)
≥4		32 (3.7)
Resilience		44.16 ± 10.17
Emotional problems		1.33 ± 1.27

**Table 2:** Bivariate correlations among the main variables.

	1	2	3	4	5	6
1 Age	--					
2 Gender	−0.028	--				
3 SES	0.005	−0.043	--			
4 ACEs	0.038	0.027	−0.088 **	--		
5 Resilience	0.059	0.074 *	0.198 **	−0.136 **	--	
6 EP	−0.051	0.001	−0.014	0.135 **	−0.276 **	--

Note: SES = socioeconomic status, ACEs = adverse childhood experiences, EP = emotional problems. * *p* < 0.05, ** *p* < 0.01.

**Table 3:** Mediating effect of resilience on ACEs and preschoolers’ emotional problems.

Predictors	Model 1 (Criterion Resilience)		Model 2 (Criterion EP)	
	b	t	b	t
CO: Age	0.16	1.99 *	−0.01	−1.17
CO: Gender	1.77	2.65 **	0.05	0.58
CO: SES	2.48	5.77 ***	0.08	1.49
X: ACEs	−1.20	−3.77 ***	0.12	3.14 **
ME: Resilience			−0.03	−8.10 ***
R^2^	0.06		0.09	
F	15.04 ***		17.18 ***	

Note. * *p* < 0.05, ** *p* < 0.01,*** *p* < 0.001. SES = socioeconomic status, ACEs = adverse childhood experiences, EP = emotional problems.

## Data Availability

Not applicable.
